# Reduction of Motion Artifacts and Improvement of R Peak Detecting Accuracy Using Adjacent Non-Intrusive ECG Sensors

**DOI:** 10.3390/s16050715

**Published:** 2016-05-17

**Authors:** Minho Choi, Jae Jin Jeong, Seung Hun Kim, Sang Woo Kim

**Affiliations:** 1 Department of Creative IT Engineering and Future IT Innovation Laboratory, Pohang University of Science and Technology (POSTECH), Pohang, Kyungbuk 790-784, Korea; minho17@postech.ac.kr; 2 Department of Electrical Engineering, Pohang University of Science and Technology (POSTECH), Pohang, Kyungbuk 790-784, Korea; jin03jin@postech.ac.kr (J.J.J.); pisics@postech.ac.kr (S.H.K.)

**Keywords:** electrocardiogram, R peak, heart rate, motion noise, active noise cancellation, adaptive filter, non contact measurement

## Abstract

Non-intrusive electrocardiogram (ECG) monitoring has many advantages: easy to measure and apply in daily life. However, motion noise in the measured signal is the major problem of non-intrusive measurement. This paper proposes a method to reduce the noise and to detect the R peaks of ECG in a stable manner in a sitting arrangement using non-intrusive sensors. The method utilizes two capacitive ECG sensors (cECGs) to measure ECG, and another two cECGs located adjacent to the sensors for ECG are added to obtain the information on motion. Then, active noise cancellation technique and the motion information are used to reduce motion noise. To verify the proposed method, ECG was measured indoors and during driving, and the accuracy of the detected R peaks was compared. After applying the method, the sum of sensitivity and positive predictivity increased 8.39% on average and 26.26% maximally in the data. Based on the results, it was confirmed that the motion noise was reduced and that more reliable R peak positions could be obtained by the proposed method. The robustness of the new ECG measurement method will elicit benefits to various health care systems that require noninvasive heart rate or heart rate variability measurements.

## 1. Introduction

The electrocardiogram (ECG) is a set of voltage signals that is generated by heart activity [1]. The R wave of ECG can be used to calculate the heart rate (HR) and its variability (HRV), which contain important information on the body’s physiological condition. This data can be used to diagnose diseases like multiple sclerosis, stroke, ischemic heart disease, and myocardial infarction, or can also be used to provide information on the autonomic nervous function [2,3]. Therefore, measuring HR or HRV in daily life can be beneficial to humans, because it can serve as a regular check of cardiac electrical activity. In addition, if an ECG system can measure the HR or HRV of a human in a vehicle during his driving route, it can detect abrupt possible cardiac abnormalities or physical changes in the driver, such as fatigue, drowsiness, and stress [4,5]. This can make driving safer and provide comfort to drivers by making it possible to take proper action in advance or by providing the necessary information when an abnormal condition is detected. However, the conventional ECG measurement method is not practical for use in daily life. It requires Ag-AgCl electrodes that must be attached to the body of the tested subjects directly. This method is rather uncomfortable and disturbs daily activities or driving.

Recently, based on advances in sensor technology and an increase in public interest in health care, a method measuring the ECG using non-intrusive electrodes was developed. This method uses capacitive ECG sensors (cECGs) as the electrodes for non-intrusive ECG measurements. The cECG measures ECG by sensing subtle changes in voltage using capacitive couplings near the human body. Therefore, this method does not require direct contact or resistive coupling with the skin. Many research projects that have applied the cECG in daily life have been conducted [6,7,8,9]. In addition, there have been some trials that used the cECG to measure ECG during driving [10,11]. The non-intrusive measurement method does not restrict a subject, so it is easy to measure the ECG and can be proven to be advantageous for long-term measurements [12]. The fact that there is no skin irritation is also one of its advantages [13]. However, the non-intrusive measurement method is very sensitive to the noise from motion. Much noise can be generated by the motion of a subject or by the swinging patterns of a vehicle when the measurement is conducted during driving. In addition to noise from motion, there can be many other noise sources in a measured ECG: baseline wander noise from respiration, power line noise, and electromyography noise from muscular movement. Among these, however, motion noise is the most difficult noise to remove because it occurs randomly and its amplitude is significant when compared with the original ECG.

Numerous studies have been conducted in order to study ways to reduce motion noise [14,15,16,17,18,19,20,21,22]. The simplest way is the application of a band-pass filter (BPF). However, the conventional BPF is not effective in reducing motion noise, because the noise has a frequency spectrum that overlaps with the ECG spectrum [14]. Wavelet transform-based methods can be used to reduce the noise in the ECG [15]. However, this transform also has a limitation owing to the large amplitude and irregular characteristics of motion noise. When several sensors are available, sensor fusion and independent component analysis (ICA)-based methods can be used [16,17]. The sensor fusion selects a sensor signal having the highest quality. However, the sensor fusion can not remove the noise itself. In case of ICA, high computational complexity is the shortcoming. Therefore, it is difficult to use ICA in a near-real-time system. Recently, the method using an injection signal was introduced [20]. It estimates the artifacts using the injection signal and the model of the capacitive system, but more investigation on real-life data seems to be needed. The extended Kalman filter (EKF) uses a nonlinear model of ECG signal and tries to apply the model to the Kalman filter that is modified to deal with nonlinear relations. EKF was studied to reduce the noise of conventional contact ECG in other papers, and it was also used for non-intrusive ECG [21,22]. Although it was useful for many cases, it can not be effective to reduce motion noise in non-intrusive ECG because of the model-based approach of EKF. In addition, some prior publications reported on the use of active noise cancellation (ANC), including adaptive filters, to reduce motion noise [18,19]. The adaptive filter can change its filter characteristics using feedback loops [23]. Therefore, it is suitable for dealing with motion noise that has time-variant characteristics. However, to reduce the noise effectively, the ANC needs a reference signal that is highly correlated with the noise and uncorrelated with the original ECG. A prior research used accelerometer data as the reference signal for the ANC, but the result was not effective [24]. The accelerometer data did not satisfy the condition needed in terms of the reference signal, whereby the correlation between the accelerometer data and the noise was not high.

A new method is proposed in this paper to overcome this problem and in an effort to obtain reliable R peak positions. The proposed method places additional cECGs adjacent to the cECGs used for ECG measurement. Then, the difference between the signals by the additional cECGs and the signals by the cECGs to measure ECG is used to construct a reference signal for ANC. For the weight updating algorithm of the adaptive filter in the ANC, affine projection algorithm (APA) is used. Post-processing is added to correct errors that can be caused by ANC in exceptional situations. In addition, the relationship of the proposed reference signal and motion noise is analyzed in the discussion section. To verify the effectiveness of the proposed method, the ECG is tested based on indoor measurements and measurements during driving. The R peak detecting rates of the ECGs before and after the application of the proposed method are then compared. The results will show that the R peaks can be detected in a more stable manner using the proposed method. Furthermore, because its computational complexity is not high, the near-real-time monitoring of HR or HRV by the detected R peaks is possible using this method.

## 2. Materials and Method

### 2.1. Measurement System

In conventional works, two or more cECGs were used as electrodes, and they were attached to a chair or a driver’s seat to measure the ECG during daily life activities or driving [25,26]. Furthermore, conductive fabric or an additional cECG was placed on the bottom of the seat for the reduction of the common noise component of the measured signals using a driven-right-leg circuit (DRL) [27].

**Figure 1:**
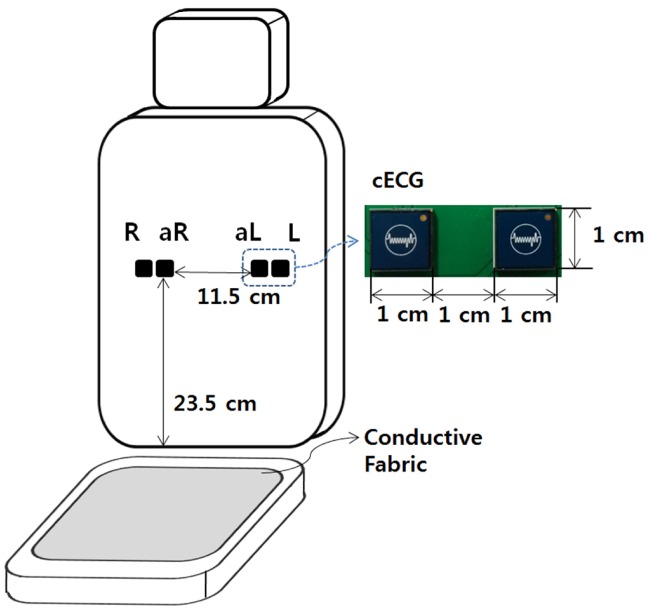
Schematic diagram of ECG measurement system placed in a seat.

Our measurement system is designed based on these prior designs. It has two cECGs attached to a seat without any covers, and the conductive fabric is placed on the bottom of the seat for the DRL. However, in our system, an additional cECG is added near the each cECG used to sense ECG as shown in Figure 1. This is to measure the motion information around the cECG for ECG measurement and to obtain a reference signal for ANC. In the figure, *R* and *L* are the right and left sensors for ECG measurement, and aR and aL are additional right and left sensors to get the motion information. The signals measured by the four cECGs are filtered using a low-pass filter with a 40 Hz cutoff frequency to remove high-frequency noise. They are then sampled to digital signals at 360 Hz through an NI 9205 from National Instruments using 16 bits for analog-digital conversion. Let the signal measured by cECG *i* be $sig_{i}$. The measured ECG ($ECG_{m}$) is obtained using the limb lead 1 method as

(1) $$ECG_{m}=BPF\left[sig_{L}-sig_{R}\right]$$

where BPF means the use of a BPF with a 0.05–35 Hz cutoff frequency [28]. In addition to these two signals, $r_{L}$ and $r_{R}$ are calculated as

(2) $$r_{L}=sig_{L}-sig_{aL}$$

(3) $$r_{R}=sig_{R}-sig_{aR}$$

They are used to construct the reference signal for ANC, and its detailed description will be presented in Section 2.3, which discusses the ANC using the reference signal.

### 2.2. Data Acquisition

Both indoor and outdoor experiments were conducted for data acquisition. In indoor experiment, our measurement system was installed in a chair where subjects sat to measure the ECG. The subjects were one 29-year-old, two 25-year-old, and one 28-year-old healthy males with no heart-related medical history. All subjects wore short sleeved t-shirts made of 100% cotton during the experiments. Five-minute ECG recordings were acquired, three times per subject. The subjects randomly moved their bodies to make a significant amount of artificial motion noise during the experiment. The reason for this motion noise is to verify the performance of our system in a severe environment. In addition to our measurement system, conventional contact electrodes were also used to obtain the true R peak position.

For the outdoor experiment, our system was integrated in the driving seat of a vehicle, and the ECG was measured for six people for a period of 15 min for each, during driving. The subjects were healthy males aged 27–32. Among them, subject 1, a male aged 29, and subject 6, a male aged 28, also participated in the indoor experiment as subject 1 and 4. The subjects wore same clothes used in the indoor experiment. The driving course was a road network that surrounds Pohang University of Science and Technology. Its total length is approximately 2 km, and it includes corners, an uphill grade of 311 m, downhill roads of 120 m, and eight speed bumps, in addition to straight lines. The subjects drove the course anticlockwise and repeatedly during the experiment at speeds lower than 50 km/h. The ECG was also recorded independently using conventional contact electrodes. This was to get the positions of the true R peaks, and detected peaks were inspected manually to correct any errors. The true R peaks were used to evaluate the accuracy of the tested R peaks.

All R peaks were detected by the Pan and Tompkins (PT) algorithm because it is simple and its performance has been well studied in many publications [29,30,31]. The PT algorithm uses a BPF of 5–15 Hz to remove unnecessary signal parts. Then, it applies derivative, squaring, and moving-average operations to the filtered signal. The R peaks are detected by a threshold-based method for the filtered and moving averaged signals at the algorithm.

### 2.3. ANC and Proposed Reference Signal

**Figure 2:**
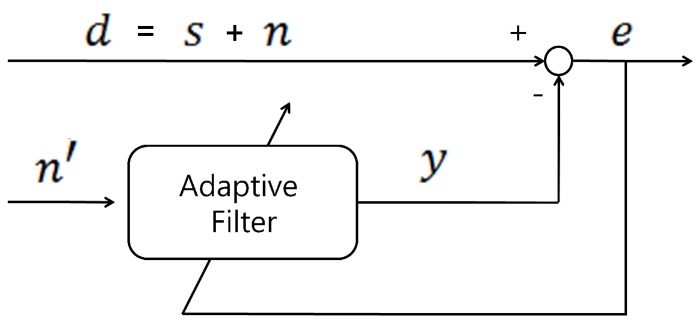
Structure of ANC used to reduce motion noise *n* in the measured ECG *d*.

Figure 2 is the basic structure of ANC [32,33,34]. In this structure, ANC separates the noise signal *n* from *d*, the sum of the original signal *s* and noise *n*, using the adaptive filter and reference signal $n^{\prime }$. For this operation, it is assumed that $n^{\prime }$ is correlated with *n* but uncorrelated with *s*. The difference between *d* and the output of the adaptive filter *y* can be expressed as the error signal *e* according to

(4) e=d−y=s+n−y

If *s* and *n* are uncorrelated and $n^{\prime }$ satisfies the former assumption, the mean square of *e* is as follows:

(5) $$\begin{array}{cc} E[e{\;}^{2}] & =E[{\left(s+n-y\right)}^{2}]\\ & =E\left[s{\;}^{2}\right]+E\left[{\left(n-y\right)}^{2}\right]+2\cdot E\left[s\left(n-y\right)\right]\\ & =E\left[s{\;}^{2}\right]+E\left[{\left(n-y\right)}^{2}\right] \end{array}$$

The adaptive filter updates its filter weights to minimize the value of $E[e{\;}^{2}]$:

(6) $$\begin{array}{cc} \min\{E\left[e{\;}^{2}\right]\} & =\min\{E\left[s{\;}^{2}\right]+E\left[{\left(n-y\right)}^{2}\right]\}\\ & =E\left[s{\;}^{2}\right]+\min\left\{E\left[{\left(n-y\right)}^{2}\right]\right\} \end{array}$$

The filter output *y* approximates the noise *n*, and the estimated *s* can be obtained from the value of *e*. In our case, *s*, *n*, and *d* represent the original ECG, motion noise, and $ECG_{m}$, respectively. For this ANC scheme, it is important to design the proper reference signal. It must have a low correlation value with respect to the original ECG and a high correlation with motion noise.

**Figure 3:**
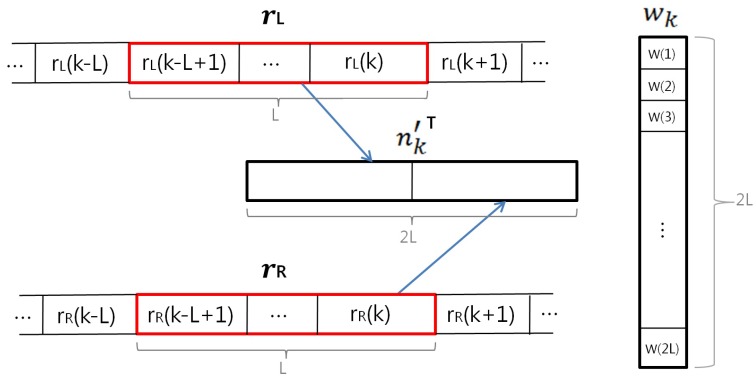
Reference signal $n_{k}^{\prime }$, and weight vector $w_{k}$ of the adaptive filter used to process the kth sample of ECG.

To obtain the reference signal that has a high correlation with motion noise, $r_{L}$ and $r_{R}$ which are constructed by the difference of adjacent cECG signals are used. This is because it is assumed that there is little difference between the cECG signals when a subject is in a static condition, and that the difference increases when the subject is in motion. The verification of its suitability as the reference signal for ANC will be analyzed in Section 4.2. To use $r_{L}$ and $r_{R}$ in our method, the two signals are combined as a reference signal $n^{\prime }$. When the length of the weight vector or tap length for the adaptive filter is 2·L, *L* elements in $r_{L}$ and $r_{R}$ are used for $n^{\prime }$, as shown in Figure 3. The tap length must be large enough to model relation between *n* and $n^{\prime }$, but too large tap length increases computational complexity. In the adaptive filter, the APA is used as the weight-updating algorithm because it converges faster than normalized least-mean-squares and it is less complex than the recursive-least-squares algorithm. Its weight vector updating equation is as follows [23]:

(7) $$\begin{array}{cc} n_{k}^{\prime } & ={\left[r_{L}\left(k-L+1\right)\;\;...\;\;r_{L}\left(k\right)\;\;r_{R}\left(k-L+1\right)\;\;...\;\;r_{R}\left(k\right)\right]}^{T} \end{array}$$

(8) $$\begin{array}{cc} U_{k} & ={\left[n_{k}^{\prime }\;\;\;n_{k-1}^{\prime }\;\;\;...\;\;\;n_{k-P+1}^{\prime }\right]}^{T} \end{array}$$

(9) $$\begin{array}{cc} d_{k} & ={\left[d\left(k\right)\;\;\;d(k-1)\;\;\;...\;\;\;d\left(k-P+1\right)\right]}^{T} \end{array}$$

(10) $$\begin{array}{cc} w_{k+1} & =w_{k}+\eta U_{k}^{T}{\left(\epsilon I+U_{k}U_{k}^{T}\right)}^{-1}\left[d_{k}-U_{k}w_{k}\right] \end{array}$$

where *η* is the step size, *ϵ* is the regularization parameter, and *I* is a *P* × *P* unit matrix. *η* determines the convergence speed of the adaptive filter. A large *η* increases the convergence speed, but it also increases misalignment. *ϵ* is set to a small constant. Then, ϵI becomes a diagonal matrix having *ϵ* as diagonal entries. It is used to prevent the case that the inverse of $U_{k}U_{k}^{T}$ does not exist in Equation (10). *P* is the projection order of APA, and it means the number of input vectors used to update the weight vector. *P* decides the convergence speed and complexity of the algorithm. A larger *P* can increase the convergence speed, but at the same time the complexity of the algorithm will be increased because the dimension of $U_{k}U_{k}^{T}$ is increased. In our method, *P* is set to 2. $w_{k}$ is the weight vector of the adaptive filter for the kth sample, and it is updated recursively. Then, the kth sample of *y* is calculated by $w_{k}$ and reference signal $n_{k}^{\prime }$ as follows:

(11) $$\begin{array}{cc} y(k) & ={n_{k}^{\prime }}^{T}\cdot w_{k} \end{array}$$

For data having *M* samples, the output of ANC ($ECG_{ANC}$) can be obtained as *e*,

(12) $$\begin{array}{cc} e(k) & =d(k)-y(k) \end{array}$$

(13) $$\begin{array}{cc} ECG_{ANC} & =e=[e(1)\;e(2)\;...\;e(M)] \end{array}$$

**Figure 4:**
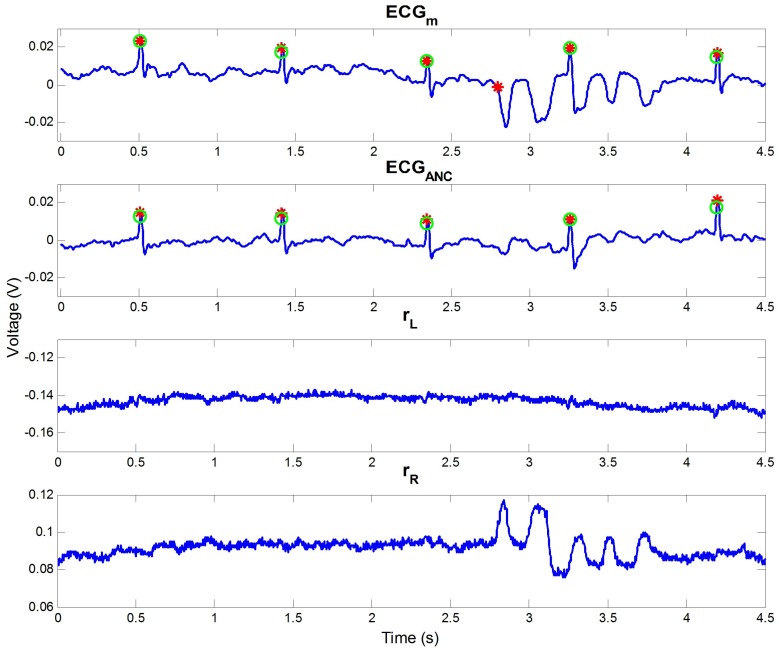
Effect of ANC, including noise reduction and the enhancement of R wave. Green circles are the true R peak positions detected by the contact ECG, and red stars are detected R peaks in each signal.

The effect of ANC can be seen in Figure 4. The figure represents a part of the data from subject 5 of our outdoor experiment. In the figure, motion noise occurred from 2.7 to 4 s of $ECG_{m}$, and the incorrect R peak was detected at approximately 2.7 s owing to the noise. If $r_{L}$ and $r_{R}$ include the motion information, the noise can be removed. In the example, $r_{R}$ was highly correlated with the motion noise. According to this, the noise could be reduced, and all R peaks could be detected well after ANC, as seen in the second graph in the figure.

### 2.4. Post-Processing

**Figure 5:**
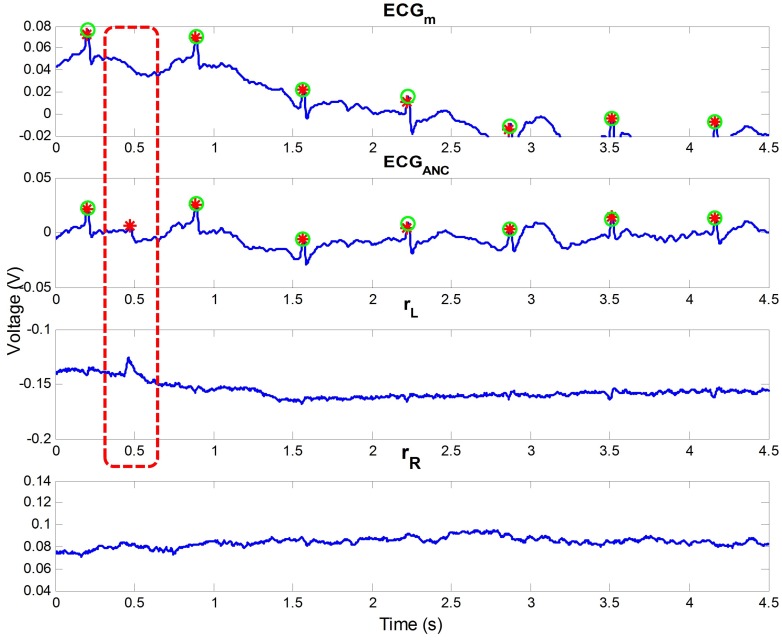
Example of the case where ANC is not effective.

Even though the proposed reference signal is effective in most cases of motion noise, exceptional situations could happen under real circumstances. These phenomena mainly occur when the additional sensor includes a noise component that does not appear in the $ECG_{m}$. That is, if a measurement or subtle motion noise occurs only at the additional sensors for the reference signal, the reference signal will contain a noise component that is uncorrelated with noise components in the $ECG_{m}$. This could cause an error when ANC is conducted because the assumption of high correlation between the reference signal and a noise component in the $ECG_{m}$ is not satisfied. Figure 5 shows an example. In the figure, a noise that is not included in the $ECG_{m}$ was measured by $r_{L}$ at 0.5 s. By this noise component, a peak was wrongly detected after ANC, and the result became more erroneous than that of the $ECG_{m}$. Therefore, for a robust and practical algorithm, this phenomenon must be prevented, and a post-processing is needed to correct the erroneous result. In our method, $ECG_{ANC}$ and $ECG_{m}$ are evaluated by two rules that are related to the change of peak interval and signal power. When $ECG_{ANC}$ is considered to be abnormal by the rules, $ECG_{ANC}$ is not used and is replaced by $ECG_{m}$. This process can be considered as a selection process to decide whether $ECG_{ANC}$ is used or not.

**Figure 6:**
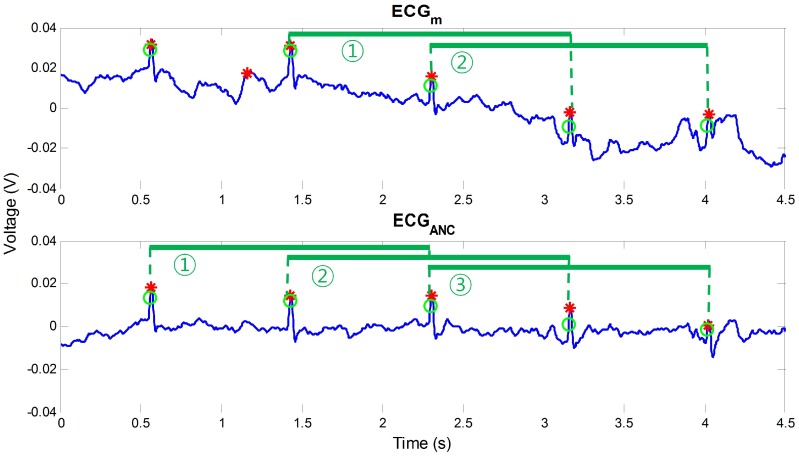
Evaluation of $ECG_{ANC}$ and $ECG_{m}$ using the inequality related to the normality of instantaneous HR.

To test two signals, the first rule checks the normality of detected R peaks. For this, the R peaks of each signal are detected by the PT algorithm mentioned in Section 2.2. Then, the validity of detected R peaks is tested by the standard to check whether the range of acceleration or deceleration of instantaneous HR is normal. The standard uses an inequality introduced in [35]:

(14) $$2\cdot \left|\frac{t_{i}-2t_{i+1}+t_{i+2}}{\left(t_{i}-t_{i+1}\right)\left(t_{i}-t_{i+2}\right)\left(t_{i+1}-t_{i+2}\right)}\right|<0.5$$

where $t_{i},\;t_{i+1},\;t_{i+2}$ are the temporal positions of the three successive R peaks. This is based on the band-limited characteristics of the variation of R peaks, and its effectiveness was verified by an open ECG database [35]. Using this standard, if the number of three peaks that satisfy the standard in $ECG_{ANC}$ is higher than that of $ECG_{m}$, then $ECG_{ANC}$ is used as the output of our algorithm. This is because it can be considered that $ECG_{ANC}$ contains more valid information on R peaks than $ECG_{m}$. As an example, only two groups of three R peaks satisfied the standard in $ECG_{m}$, as shown in Figure 6. On the other hand, the number of three R peaks groups that satisfy the standard was three for $ECG_{ANC}$ by reduced noise. In this case, $ECG_{ANC}$ is selected as the output of the algorithm by the rule.

For cases that do not satisfy the first rule, the second rule is applied. The rule compares the change of signal power in 5–15 Hz after ANC. The range of 5–15 Hz is the frequency band of an R peak [29]. Therefore, if ANC operates effectively and motion noise is reduced, the power of the frequency band will be decreased. This is because ANC assumes that the measured signal contains an additive noise to an original signal, and ANC operates to cancel out the noise. On the other hand, the increased power of the frequency band can be considered as an abnormal operation of ANC. As seen in Figure 5, if the reference signal contains noise that is uncorrelated with $ECG_{m}$, it can generate additional noise that can cause the incorrectly detected R peaks or a performance degradation in R peak detection. For this reason, $ECG_{ANC}$ is discarded and $ECG_{m}$ is used as the output of the proposed algorithm when this rule is satisfied:

(15) $$P_{ANC}>P_{m}$$

where $P_{ANC}$ is the 5–15 Hz signal power of $ECG_{ANC}$ and $P_{m}$ is that of $ECG_{m}$. For the rest of the cases, $ECG_{ANC}$ is used as the output of the algorithm.

## 3. Experimental Results

### 3.1. Performance Index

**Figure 7:**
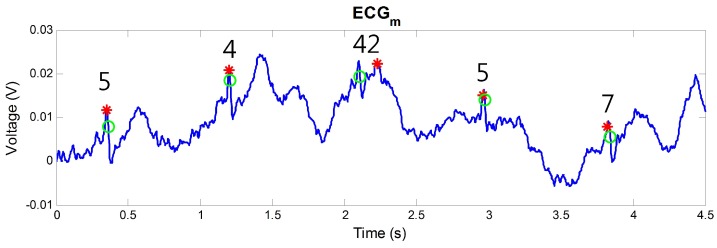
Detected R peaks in ECG measured by cECG. The numbers on the detected R peaks represent the sample difference with each true R peak positions.

To represent the accuracy of the detected R peaks, two performance indices were used: a) the sensitivity (Se), and b) the positive predictivity ($P^{+}$) index. The two indices are calculated as follows,

(16) $$Se=\frac{TP}{TP+FN}\times 100$$

(17) $$P^{+}=\frac{TP}{TP+FP}\times 100$$

where TP is the number of correctly detected R peaks, FN is the number of undetected R peaks within searching windows based on true R peaks, and FP is the number of detected R peaks at the outside of the searching window. Conventionally, 150 ms of searching window is used and it has 54 sample points in our system with a 360 Hz sampling rate [36]. However, we used 15 sample points for the searching window to identify the correctly detected R peaks because the ECG measured in the non-intrusive manner is much noisy than the ECG by the contact electrodes. Figure 7 shows the necessity of the decreased searching window. In the figure, the third peak was incorrectly detected by noise, but the sample difference with the position of its true R peak was 42 samples. In this case, the peak can be considered as the correctly detected R peak by the 150 ms of searching window. To avoid the false classification, the decreased searching window was used.

### 3.2. Comparison of Results

To verify the effect of the proposed method, we applied the method to the experimental data. In this process, every 4.5 s block of recorded data were processed sequentially. We used the 4.5 s length because at least three R peaks were needed for our post-processing. More longer block can be used, but we did not enlarge the length considering near-real-time operation. Furthermore, short block length will be advantageous for the post-processing because signal can be evaluated more minutely. In the processed data, 1.5 s of data represented the overlapped data that were used in previous processing. The overlapped data were needed to detect a R peak occurred at the boundary of two processing windows stably. The step size and tap length of the adaptive filter were set to 0.01 and 360 experimentally.

A comparison of the results for the indoor and outdoor experiments is listed in Table 1 and Table 2. In the tables, Se and $P^{+}$ are presented for $ECG_{m}$, $ECG_{ANC}$, and the signal after post-processing. To show the difficulty of using a conventional ECG denoising method for the signal by a non-intrusive sensor, the performance indices of a wavelet-thresholding-based method were attached together [37]. In addition, the results by EKF were presented for comparison. In the table for the indoor experiment, data 1-1, 1-2, and 1-3 are the data from the subject 1. Likewise, data 2-1, 2-2, and 2-3, and data 3-1, 3-2, and 3-3, and data 4-1, 4-2, and 4-3 were measured from same subjects. d_acc is the difference of $Se+P^{+}$ between the tested signal and that of $ECG_{m}$. That is, let Se and $P^{+}$ of $ECG_{m}$ be $Se_{m}$ and $P_{m}^{+}$, and those of the tested signal be $Se_{t}$ and $P_{t}^{+}$. Then, d_acc is as follows:

(18) $$d\_acc=\left(Se_{t}+P_{t}^{+}\right)-\left(Se_{m}+P_{m}^{+}\right)$$

This is calculated to compare the increase in R peak detecting accuracy by applied methods considering both Se and $P^{+}$.

In Table 1, the effect of ANC could be identified. For most data, Se and $P^{+}$ were increased after ANC. However, the R peak detecting accuracy was lower than that of $ECG_{m}$ at data 3-3. This was because the reference signal of the data contained uncorrelated noise with the $ECG_{m}$, as seen in Figure 5. This noise could happen by an electrical or measurement noise at the additional sensors used to construct the reference signal. Post-processing is added for cases like this. After post-processing, the accuracy of all data were higher than that of the $ECG_{m}$. $Se+P^{+}$ increased 8.91% on average and 26.26% maximally at data 2-1. For the outdoor data, the proposed method likewise detected R peaks more accurately, as seen in Table 2. The proposed method increased $Se+P^{+}$ by 7.36% on average. However, ANC itself was not effective and the increase in accuracy was not significant after post-processing for outdoor data 1, 2, and 3. This was related to the characteristics of the data and will be discussed in the next section.

Post-processing increased the accuracy in most data. Its effect was noticeable at indoor data 3-3, and outdoor data 1, 2, and 3 having lower accuracy than $ECG_{m}$ after ANC. By the post-processing, the accuracy of the data was increased and it was higher than that of $ECG_{m}$ likewise other data. On the other hand, the R peak detecting accuracy of ANC decreased for indoor data 2-1, 3-2, and 4-2 after the post-processing. This was because the post-processing could not perfectly distinguish the signal having better accuracy in a certain processing window. It was hard to choose better signal when the signal contained a noisy or irregular component. However, the decreased accuracy was insignificant and it was lower than 0.5%. Furthermore, the accuracy increased for all outdoor data having longer data length than indoor data after the post-processing. This result shows an advantage of the post-processing for general cases.

**Figure 8:**
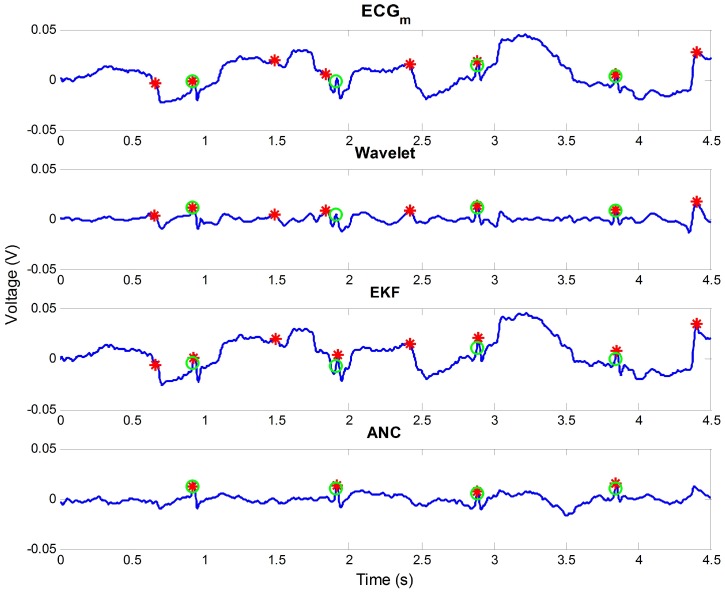
Comparison of ANC with other methods for a signal part in outdoor data 5.

In the results, the wavelet-based method had an effect on the indoor data 1-3, 2-2, 3-2, 4-2, and 4-3 and outdoor data 1, 2, and 6, but the accuracy was mostly lower than the original one. This was because of the large amplitude of motion noise. The wavelet-based method divides the signal into the signals of a certain frequency band, and it applies a threshold to remove noise components. Therefore, this method was not effective in the non-intrusive ECG because the ECG contained motion noise having a large amplitude. The EKF enhanced the R peak detecting accuracy of all data except for indoor data 1-1 and outdoor data 3. For indoor data 3-3 and 4-3, its accuracy was higher than that of ANC with post-processing. However, the increased accuracy was not significant for all data. This limitation comes from the model-based approach of EKF. It uses an ECG model to estimate the original ECG, but the used model will have a large error with a measured signal. This error can be generated by the low signal-to-noise ratio of the non-intrusive ECG signal itself and the motion noise having a significant amplitude. The increased $Se+P^{+}$ was maximum in indoor data 1-3 as 2.59% by the EKF. On the other hand, the effect of ANC was remarkable. Its accuracy was higher than other methods for most of the data, and its increased range was significant. Furthermore, it could improve the accuracy of all data with post-processing. The averaged rate of increase was 8.39% for all experimental data. Figure 8 shows one example that represents the advantage of ANC. The wavelet-based method and EKF were not effective to reduce motion noise in the figure. However, ANC could reduce the motion noise prominently and all R peaks were correctly detected. This effectiveness of ANC results from the proposed reference signal that contains information on the occurred motion noise.

In addition, the complexity of our method is not high. In our experiments, the measured ECG was processed by MATLAB and a personal computer having an Intel Core i7-3930k CPU and 16 GB of RAM. The processing time for the data divided into a 4.5 s length was 0.21 s on average and total processing time for the entire data including total indoor and outdoor data was 662.98 s. In the 4.5 s data, a 1.5 s length is overlapped data and 3 s is newly processed data. The 0.21 s is very short compared with the 3 s length of the new data. Therefore, our algorithm could be implemented in a near-real-time system.

## 4. Discussion

### 4.1. Analysis on Outdoor Data

In Table 2, ANC was less effective for outdoor data 1, 2, and 3 compared to indoor data. To investigate the reason, the outdoor data were analyzed in Table 3. In the table, Se+$P^{+}$ represents the sum of Se and $P^{+}$ for the each $ECG_{m}$ of outdoor data. Se+$P^{+}$ was high in outdoor data 1, 2, and 3 because the data had little noise. In this case, the application of ANC could not make much difference in R peak detecting accuracy because the signal was already clean and contained little noise. This can be one reason that the effect of ANC was not remarkable.

To know the characteristics of noise in the data, we divided the processing windows of each outdoor data into two groups. One is the windows having 100% of Se and $P^{+}$. The windows can be considered signal parts having little noise, and the averaged signal power of the windows ($P_{clean}$) was calculated to compare it with noise power ($P_{noise}$) in each data. Windows in the other group can be considered as noisy signal parts because it contains R peak detecting error. $P_{noise}$ was obtained as the averaged signal power of the windows in each data, and the relative amplitude of $P_{noise}$ ($r\_P_{noise}$) for clean signal parts was calculated as

(19) $$\begin{array}{c} r\_P_{noise}=\frac{P_{noise}}{P_{clean}} \end{array}$$

$r\_P_{noise}$ was extremely high in outdoor data 2 and 3 as seen in Table 3. This severe noise occurred when the body of subjects was totally detached and far away from the cECGs by the manipulation of the steering wheel in a corner, or by the sway of the vehicle owing to speed bumps. For this excessively severe noise, ANC was not effective. This is because additional cECGs could not measure the valid motion information that was related to the noise in $ECG_{m}$. Furthermore, the characteristics of noise measured in the additional cECGs could be much different from the noise in $ECG_{m}$. Outdoor data 2 and 3 included a small number of noisy windows, and the noise of the windows was severe. The use of ANC was not effective in the data by this reason. When the data were measured, the movement of subjects was restricted by the cautious behavior of subjects for the experiment and the use of contact ECG electrodes. This could make the data contain a few signal parts having the noise that can be effectively reduced by ANC. In real driving situations where the various movements of a subject occur, the effect of ANC will be more prominent as in the rest of the outdoor data.

**Figure 9:**
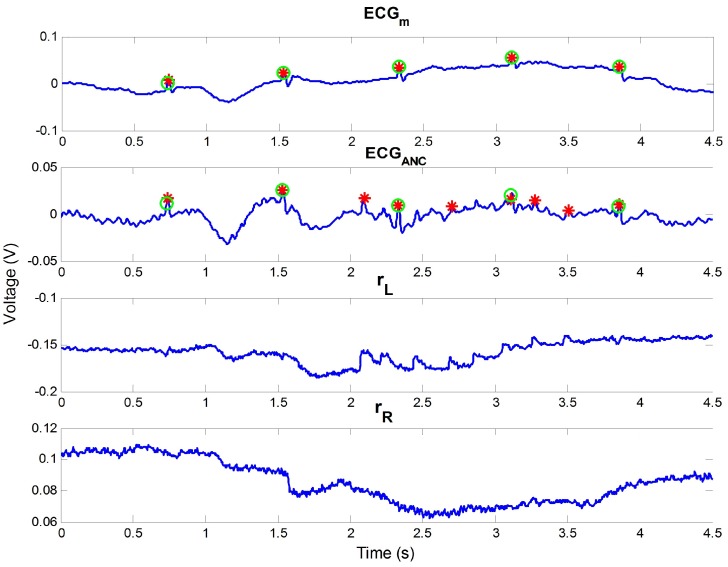
Erroneous case of ANC for a signal part in outdoor data 1.

In addition, the same problem with indoor data 3-3 occurred in some outdoor data as seen in Figure 9. This problem was caused by the motion information that is included in reference signal but uncorrelated with motion noise in $ECG_{m}$. A measurement noise or electrical noise can be a reason for this phenomenon. However, these errors were corrected by post-processing. After ANC with the post-processing, R peak detecting accuracy was increased, and the increased accuracy outperformed those of other methods in all outdoor data.

### 4.2. Appropriateness of Proposed Reference Signal

**Figure 10:**
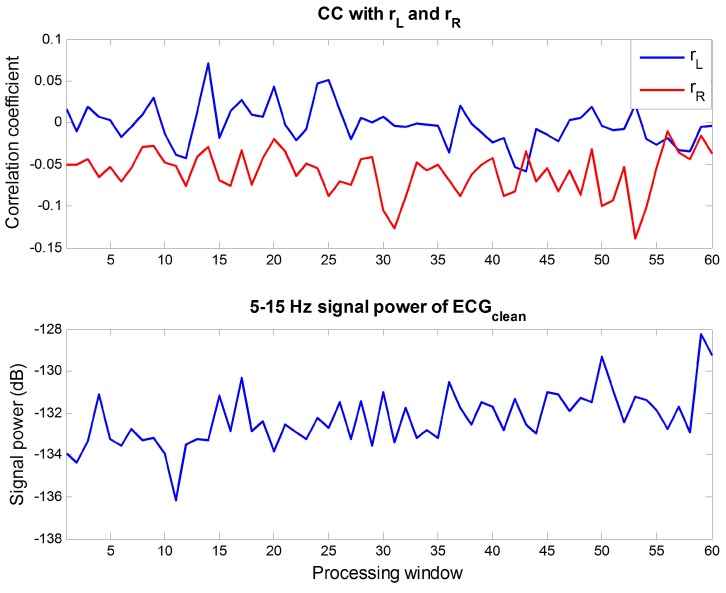
(**Top**) CCs between the 5–15 Hz components of $ECG_{clean}$ and $r_{L}$, $r_{R}$; (**Bottom**) 5–15 Hz signal power of $ECG_{clean}$ at each processing window.

**Figure 11:**
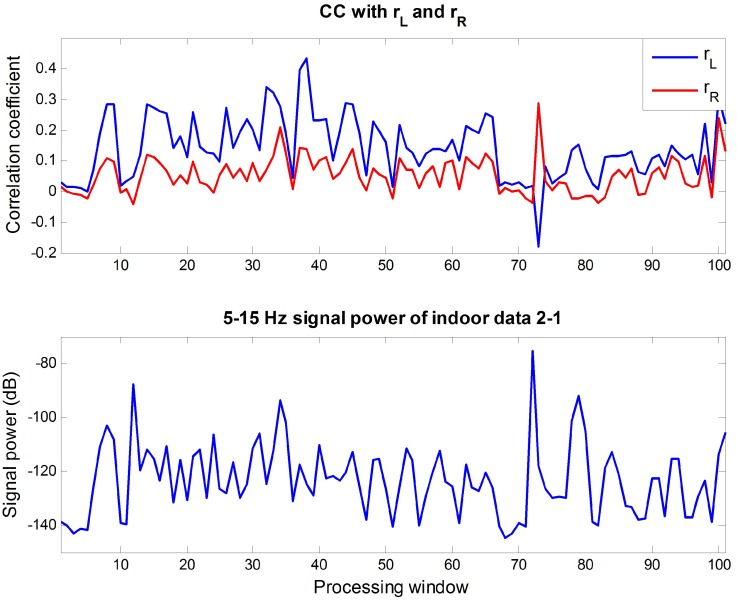
(**Top**) CCs between the 5–15 Hz components of indoor data 2-1 and $r_{L}$, $r_{R}$; (**Bottom**) 5–15 Hz signal power of indoor data 2-1 at each processing window.

This section analyzes the suitability of the proposed reference signal as a reference signal for ANC. First, to confirm the assumption that the reference signal and the R wave of the ECG are uncorrelated, the ECG with a 3-min length ($ECG_{clean}$) was measured in a stable condition to make it contain only the ECG itself with as little motion noise as possible. Then, $ECG_{clean}$ passed through the BPF of 5–15 Hz to get R waves, and the correlation coefficients (CC) with $r_{L}$ and $r_{R}$ constructing the reference signal were calculated. For two representative signals *X* and *Y*, having *M* samples each, their correlation can be expressed by CC:

(20) $$CC=\frac{\sum_{i=1}^{M}\left(X_{i}-\bar{X}\right)\left(Y_{i}-\bar{Y}\right)}{\sqrt{\sum_{i=1}^{M}{\left(X_{i}-\bar{X}\right)}^{2}}\sqrt{\sum_{i=1}^{M}{\left(Y_{i}-\bar{Y}\right)}^{2}}}$$

where $\bar{X}$ and $\bar{Y}$ are the means of *X* and *Y* [38]. If the two signals have a low correlation, the value of CC will be close to 0. For signals that have a high correlation, the absolute value of CC will be close to 1. For the 5–15 Hz components of $ECG_{clean},$ calculated CC can be seen in Figure 10, and the same analysis was conducted for indoor data 2-1 in Figure 11 for comparison. The lower parts of two figures represent the 5–15 Hz signal power of each signal. The signal power was much higher in the indoor data 2-1 because it contained much motion noise. Looking at the results on CC, the absolute value of CC was lower than 0.15 when there was little motion noise and the signal was approximated to the R wave. Comparing this result with the CC of the noisy signal in Figure 11, it is obvious that the correlations between R wave and $r_{L}$, $r_{R}$ are low. Therefore, it can be concluded that the correlation between the proposed reference signal and the R wave is low because the proposed reference signal consists of $r_{L}$ and $r_{R}$.

From the former results, the correlation between motion noise and $r_{L}$, $r_{R}$ could also be known because the absolute value of CC was increased when the motion noise occurred. To show a more generalized result, the $ECG_{m}$ of the indoor data and $ECG_{clean}$ were filtered by a HPF of 1 Hz to reduce the effect of respiration. Let the filtered ECG be $ECG_{H}$, the sum of absolute values of CCs between $ECG_{H}$ with $r_{L}$ and with $r_{R}$ ($CC_{L+R}$) was calculated at every processing window unit. Then, $CC_{L+R}$s were averaged for each indoor data and $ECG_{clean}$. That is, the averaged $CC_{L+R}$ ($a\_CC_{L+R}$) is calculated as

(21) $$\begin{array}{cc} CC_{L+R,i}\; & =abs\left(CC_{L,i}\right)+abs\left(CC_{R,i}\right) \end{array}$$

(22) $$\begin{array}{cc} a\_CC_{L+R}\; & =\frac{1}{N}\sum_{i=1}^{N}CC_{L+R,i} \end{array}$$

where *N* is the number of processing windows in each data. $CC_{L,i}$ or $CC_{R,i}$ is the CC between the tested $ECG_{H}$ and $r_{L}$ or $r_{R}$ in the ith processing window. The calculated $a\_CC_{L+R}$ is listed in Table 4. In all cases, $a\_CC_{L+R}$ of indoor data containing motion noise was higher than $ECG_{clean}$. This result reveals that the reference signal and the motion noise have a correlation because $a\_CC_{L+R}$ increased when the ECG contained the motion noise.

**Figure 12:**
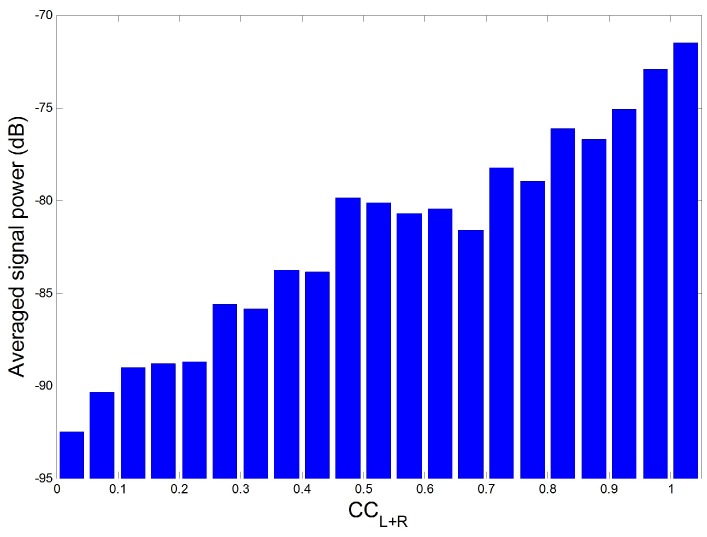
Relation between $CC_{L+R}$ and averaged signal power.

To validate the relation in more detail, all $ECG_{clean}$, indoor and outdoor data were filtered by the HPF of 1 Hz and the averaged signal power was calculated according to $CC_{L+R}$. That is, $CC_{L+R}$ was divide into 0.05 interval and signal power in each interval was averaged in Figure 12. For the experiment, a processing window having signal power higher than a threshold was treated as a outlier and excluded. The threshold was set as 100 times $P_{clean}$ for each data. $P_{clean}$ is obtained as the averaged signal power of processing windows having 100% of Se and $P^{+}$ for each data as mentioned in the previous section. In the Figure 12, the degree of correlation between the reference signal and ECG was increased mostly in accordance with the increase in signal power or the occurrence of motion noise. This tendency can be considered as the evidence of a high correlation between the reference signal and the motion noise because the correlation is increased as the ratio of motion noise in ECG increased.

## 5. Conclusions

This study proposes a method to reduce motion noise and to accurately detect R peaks using non-intrusive sensors. It uses additional cECGs placed adjacent to the cECGs for ECG measurement. Then, a reference signal is constructed using the sensor signals, and it is utilized in ANC including an adaptive filter with an APA to reduce motion noise and enhance R peaks. Post-processing is added to prevent an incorrect result in exceptional situations and to make our method more practical. In experiments, the system was implemented in a chair and a driving seat. Based on the results, an increase in R peak detecting accuracy was verified when the proposed method was used. Then, the analysis of the proposed reference signal was conducted to show its suitability for theoretical assumptions.

The proposal for the new reference signal to denoise non-intrusive ECG is our original work. In addition, the effect of our method using the new reference signal could be shown numerically by our experimental results. Because of the advantages of our method, more accurate and stable HR or HRV values can be obtained by detected R peaks in non-intrusive measurements. In addition, the proposed method does not need high-computational complexity, and it can proceed in near-real-time. Therefore, our method can be used in applications that require real-time HR or HRV information and that use a device having a shape that is similar to a chair. One of the examples is a driver’s condition monitoring system.

To improve our system, an adaptive filter technique like variable step size algorithms or the combination of two adaptive filters can be used to deal with motion noise. Furthermore, post-processing can be modified to obtain better results and not to degrade the performance of ANC as pointed out at result section. In addition, just one additional cECG was used in our method to obtain motion information for each left and right side, because this research was conducted to investigate its availability. Therefore, research using more sensors can be performed to improve R peak detecting accuracy in the future. Moreover, the system was integrated with a chair and driving seat in our experiment. Research to apply our system to a wearable device can be conducted. This will expand the applicability of our method.

## Figures and Tables

**Table 1:** Comparison results of R peak detecting accuracy for indoor data having severe motion noise.

Data	${\mathrm{ECG}}_{m}$			Wavelet				EKF				ANC				After Post-Processing		
	Se	$P^{+}$		Se	$P^{+}$	d_acc		Se	$P^{+}$	d_acc		Se	$P^{+}$	d_acc		Se	$P^{+}$	d_acc
1-1	66.41	51.83		65.63	51.12	−1.49		66.41	51.10	−0.73		74.22	58.28	14.27		75.00	58.90	15.66
1-2	88.51	72.84		87.53	73.36	−0.45		88.26	73.08	0.00		88.26	74.43	1.35		88.75	75.00	2.41
1-3	84.06	72.96		83.82	73.36	0.16		85.02	74.58	2.59		84.54	75.27	2.80		85.51	75.97	4.46
2-1	53.18	45.63		50.38	41.86	−6.57		54.45	46.62	2.26		68.96	56.58	26.72		68.70	56.37	26.26
2-2	65.59	58.89		66.09	61.81	3.41		66.09	60.68	2.29		72.52	66.44	14.48		72.77	66.37	14.66
2-3	65.51	58.41		64.02	56.95	−2.94		67.25	58.53	1.86		69.73	60.17	5.98		69.98	61.17	7.23
3-1	84.84	72.59		84.11	72.73	−0.60		85.82	73.28	1.66		90.95	80.87	14.39		90.95	81.40	14.92
3-2	89.39	78.32		89.39	78.49	0.17		89.14	79.15	0.58		92.17	79.52	3.98		91.67	79.61	3.56
3-3	93.35	85.61		93.09	84.95	−0.92		94.15	86.55	1.74		92.82	85.12	−1.02		93.09	86.42	0.54
4-1	86.39	72.56		85.15	71.97	−1.83		87.13	74.11	2.29		86.88	75.16	3.10		87.87	75.85	4.78
4-2	64.57	54.33		64.32	55.05	0.47		66.33	54.32	1.75		71.61	59.25	11.95		71.61	58.88	11.59
4-3	92.33	81.73		92.59	82.35	0.88		93.39	82.48	1.80		91.80	82.42	0.16		92.86	82.01	0.81
Average	77.84	67.14		77.18	67.00	−0.81		78.62	67.87	1.51		82.04	71.13	8.18		82.40	71.49	8.91

**Table 2:** Comparison results of R peak detecting accuracy for outdoor experiment.

Data	${\mathrm{ECG}}_{m}$			Wavelet				EKF				ANC				After Post-Processing		
	Se	$P^{+}$		Se	$P^{+}$	d_acc		Se	$P^{+}$	d_acc		Se	$P^{+}$	d_acc		Se	$P^{+}$	d_acc
1	94.27	87.52		94.19	88.07	0.48		94.81	87.64	0.66		92.74	85.37	−3.67		94.27	88.21	0.69
2	90.14	88.65		90.14	88.79	0.14		91.17	89.88	2.26		89.74	87.37	−1.68		91.49	89.56	2.26
3	96.59	92.86		96.50	92.86	−0.09		96.50	92.70	−0.24		96.16	92.45	−0.84		96.59	93.17	0.31
4	92.50	77.96		91.98	78.49	0.00		93.13	79.89	2.55		94.69	86.24	10.47		95.31	85.83	10.68
5	78.01	65.12		77.04	65.47	−0.62		79.68	64.81	1.35		87.07	73.99	17.93		87.07	74.49	18.43
6	84.52	78.50		84.45	78.69	0.12		85.42	78.63	1.03		90.12	81.40	8.50		90.95	83.88	11.80
Average	89.34	81.77		89.05	82.06	0.00		90.12	82.26	1.27		91.75	84.47	5.12		92.61	85.86	7.36

**Table 3:** Signal quality and noise power analysis for outdoor data.

Data	1	2	3	4	5	6
Se+$P^{+}$	181.79	178.79	189.45	170.46	143.14	136.02
$r\_P_{noise}$	3.25	67.93	65.48	4.35	9.26	7.41

**Table 4:** Averaged sum of absolute values of CCs between the $ECG_{H}$ with $r_{L}$ and with $r_{R}$ for the indoor data and $ECG_{clean}$.

Data	1-1	1-2	1-3	2-1	2-2	2-3	3-1	3-2	3-3	4-1	4-2	4-3	$ECG_{clean}$
$a\_CC_{L+R}$	0.38	0.33	0.40	0.65	0.43	0.66	0.36	0.30	0.31	0.43	0.33	0.33	0.17
